# Effect of slope position on the spatial distribution of soil preferential flow paths based on point pattern analysis

**DOI:** 10.1371/journal.pone.0315660

**Published:** 2024-12-19

**Authors:** Mingfeng Li, Jingjing Yao, Ru Yan, Yingxu Fan, Shuqin Liu

**Affiliations:** 1 College of Resources and Environmental Engineering, Anshun University, Anshun, China; 2 Institute of Resources and Environment, Beijing Academy of Science and Technology, Beijing, China; 3 Qinghai Forestry Carbon Sequestration Service Center, Xining, China; Tokai University School of Medicine: Tokai Daigaku Igakubu Daigakuin Igaku Kenkyuka, JAPAN

## Abstract

The mechanisms of soil preferential flow occurrence and development are influenced by topographic and geomorphological factors. However, the complex distribution patterns of soil preferential flow paths on different slopes remain unclear. Therefore, taking the subtropical evergreen broad-leaved forest area of Simian Mountain as the study area, this study conducted a quantitative analysis of the spatial distribution characteristics and spatial association of soil preferential flow at different slope positions (slope shoulder (SS), mid-slope (SM) and slope foot (SF)) using field staining tracer tests and spatial point pattern analysis. Results showed that the maximum infiltration depth, the depth of matrix flow, average dye coverage, preferential flow fraction and length index of the SM plot were all greater than those of the SS and SF plots. The number of preferential flow paths in different slope positions decreases with soil depth, with most preferential flow paths measuring 2.5–5.0 mm in each soil layer. The total number of preferential flow paths in SM plot was approximately twice that of the SS and SF plots, and the preferential flow paths in SM plot, regardless of pore size, were all in an aggregated distribution. These findings indicated that differences in soil physical properties and vegetation coverage at different slope positions lead to variations in the spatial distribution and association of soil preferential flow paths. Accordingly, strategies must be modified for the distribution, storage and utilisation of soil water resources. This study provides a theoretical basis for the effective management and protection of water resources in the Simian Mountain area and a scientific basis for exploring the mechanisms of the occurrence and development of the water cycle in forested watersheds.

## Introduction

Water source conservation and soil maintenance are among the primary ecological functions of forest ecosystems, with their water regulation, storage and retention functions primarily carried out by the soil layer [1, 2]. Mechanisms of rainfall regulation in the soil layer include water infiltration and storage, with varying retention capacities due to differences in soil physical and chemical properties [3]. As a structurally complex porous medium, soil has highly variable spatial characteristics that determine the complexity of water distribution and movement within it [4]. Water infiltration in the soil layer involves two forms of movement: uniform infiltration and non-uniform infiltration [5]. Non-uniform infiltration is also called soil preferential flow [6, 7].

Preferential flow refers to soil fissures formed by alternating wet and dry soil conditions, as well as by pore channels created by soil biota activity, plant root growth and decay, allowing water and nutrients to rapidly bypass or move through the soil matrix, reaching deeper soil layers and groundwater [8]. The occurrence of preferential flow diminishes the water retention capacity of the soil [9], which consequently weakens the function of the soil in regulating and storing precipitation, and further impacts the total amount of available water resources within the region. Moreover, the soil erosion issues triggered by preferential flow will not only lead to the loss of soil fertility but also may cause sediment deposition in water bodies such as rivers and lakes, thereby affecting the quality and availability of water resources and increasing the difficulty and cost of water resource management [10, 11].

Furthermore, the occurrence of soil preferential flow has been found to enhance the migration of organic substances such as soil nutrients, chemical fertilizers, and pesticides [12]. This not only has been disrupting the inherent ecological balance of the soil, leading to soil impoverishment and degradation, but also, once these pollutants enter water bodies, they have been posing a serious threat to the living environment of aquatic organisms and undermining the stability of aquatic ecosystems. The loss of freshwater resources has also been affecting the survival conditions of terrestrial animals and plants that rely on water, thereby having influenced the balance of the entire regional ecosystem. Consequently, soil preferential flow is a crucial factor influencing vegetation survival, preventing land degradation, protecting the regional water environment and ecological security, and serves as an important link in maintaining the sustainable development of ecosystems [13]. In-depth research on soil preferential flow is of vital significance for maintaining the health and stability of regional ecosystems as well as for water resource management.

The occurrence and development of preferential flow exhibit strong spatial heterogeneity, with complex influencing factors such as soil properties, topography, land-use types and hydraulic conditions affecting the generation and development of preferential flow paths [14]. In terms of topography, preferential flow is closely related to hillslope hydrological processes, reducing surface runoff, increasing subsurface flow and facilitating rapid downward movement of soil water and nutrients [15–18]. Existing research has largely clarified the factors influencing the occurrence and development of soil preferential flow. However, within the regional scale, there has been relatively little research reported on the spatial variability of soil preferential flow caused by the differences in soil texture, land use type, soil physical properties, and vegetation type induced by different slope positions. This situation has led to the difficulty in comprehensively and accurately grasping the specific characteristics and changing patterns of soil preferential flow under different slope positions, thereby causing the regulation and utilization of water resources by the soil to fail to proceed as expected and bringing numerous challenges to the rational allocation and efficient utilization of water resources within the region.

The preferential flow channels in soil, often represented as ‘preferential flow paths’, play a crucial role in the occurrence and development of preferential flow, influenced by the spatial distribution of preferential flow paths. The number, size, shape and connectivity of preferential flow paths determine the water movement speed, flux and water retention capacity of the soil [19]. Current research on the horizontal spatial distribution of preferential flow paths in soil mainly focuses on an image analysis of stained soil profiles to analyse the number, distribution and composition of preferential flow paths as well as the inter-distances between different diameter classes of preferential flow paths [20–22]. Systematic quantitative research on preferential flow paths is insufficient.

Quantitative ecology studies often utilise spatial point pattern methods to analyse various aspects of individual spatial characteristics, including quantity, distribution, composition and structural characteristics. These methods allow researchers to qualitatively or quantitatively describe the spatial distribution patterns of individuals [23]. Such analyses help researchers gain a deeper understanding of the processes and mechanisms of spatial pattern formation. Similarly, in quantitative research on soil preferential flow paths, horizontal profiles at different soil depths are viewed as spatial entities, where each preferential flow path on a profile is considered an individual distributed independently in space [24]. Therefore, spatial point pattern analysis methods can be used to explore the spatial location relationships between preferential flow paths and the spatial association in their distribution [25].

The research area was located in the subtropical evergreen broad-leaved forest of Simian Mountain in Chongqing. Field dye tracing experiments and spatial point pattern analysis were conducted to extract the characteristics of preferential flow and spatial locations of preferential flow paths in vertical and horizontal dye profiles of soil. The study quantitatively analysed the spatial distribution characteristics and spatial associations of preferential flow in different slope positions. The goal was to provide data simulation for preferential flow transport processes in different slope positions at a regional scale, aiming to offer a theoretical basis for the effective management and protection of water resources. Moreover, it was also aimed at providing a basis for formulating scientifically reasonable ecological protection measures by means of in-depth understanding of the characteristics and spatial distribution patterns of soil preferential flow.

## Materials and methods

### Field site

The study area is located in Jiangjin District, Chongqing, China, at Simian Mountain (106°17′–106°30′E, 28°31′–28°43′N), with an elevation ranging from 500 m to 1780 m. The geological foundation is mainly composed of purple-red sandstone, and the types of soil include purple, yellow and yellow-brown soil. The climate of this area is classified as subtropical humid monsoon climate, with an average temperature range of 13.6°C–18.4°C. The majority of rainfall occurs from June to August, with an annual average precipitation of 1221–1530 mm. The average annual runoff depth due to rainfall is 650–700 mm. This study area is characterised by a subtropical evergreen broad-leaved forest, with dominant tree species such as *Castanopsis fargesii*, *Lithocarpus glaber*, *Schima superba*, *Quercus acutissima*, *Cunninghamia lanceolata*, *Pinus armandii*, *Quercus acutissima* and *Phyllostachys pubescens*. The main shrubs include *Eurga loquaiana*, *Symplocos lancifolia*, *Rhododendron bachii*, *Catanopsis platyacantha*, *Rhododendron simsii* and *Maesa japonica*. The primary herbaceous plants consist of *Arachniodes exilis*, *Osmunda japonica*, *Hicriopteris chinese*, *Aster ageratoides*, *Woodwardia japonica* and *Dichondra repens*. Major crops cultivated in the area include soybeans and corn.

### Experimental design

**Fig 1:**
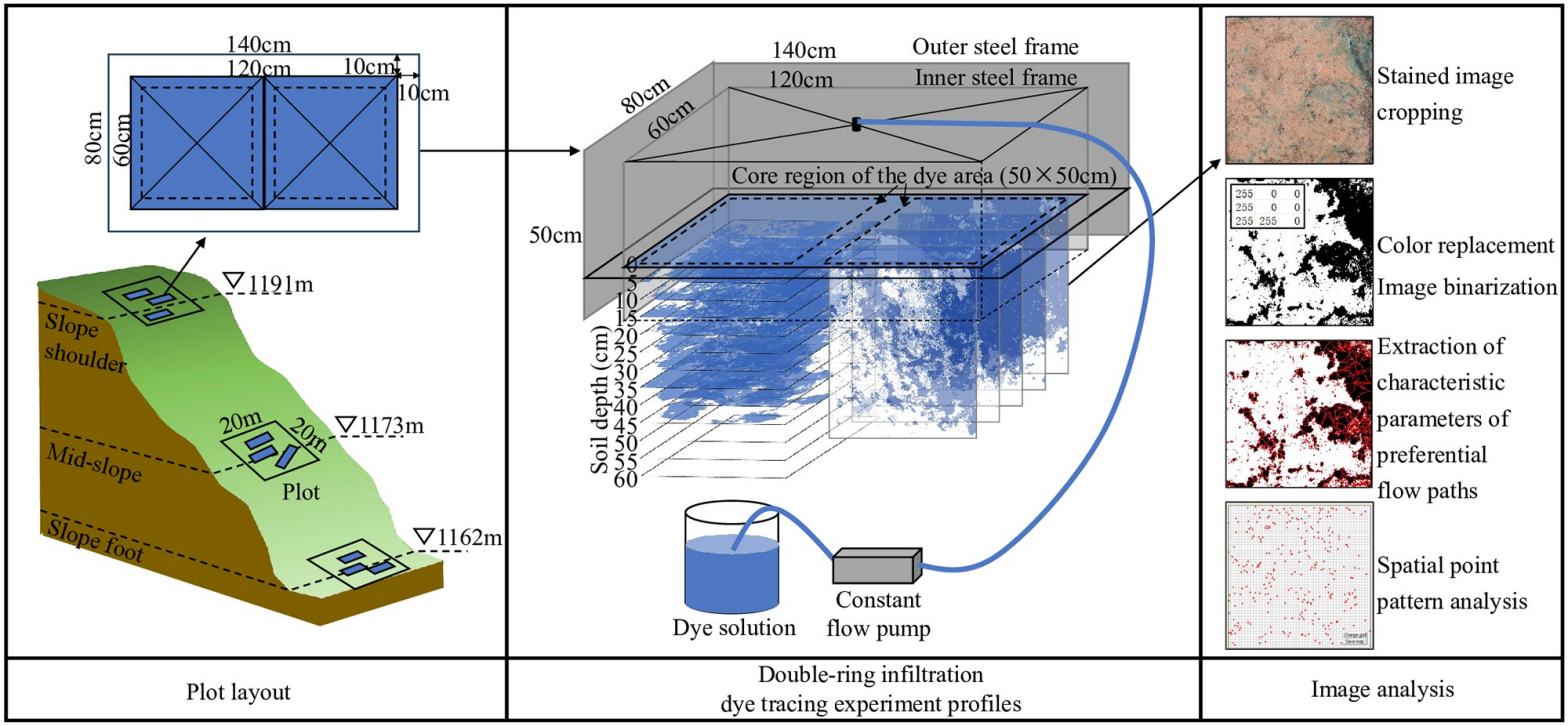
Schematic diagram of the experimental process.

Given that the study area pertains to a forest ecosystem, it is imperative to conduct a comprehensive consideration of the influence exerted by forest communities (encompassing trees, shrubs, and herbs) on soil preferential flow. A thorough investigation of the terrain, vegetation and soil elements was conducted in the study area. Within the same typical watershed, 20 × 20 m preferential flow observation plots were set up on the slope shoulder (SS), mid-slope (SM) and slope foot (SF). Within each plot, 3 double-ring infiltration dye tracing experiment profiles were randomly established, totalling 9 profiles (Fig 1).

In order to ensure that the dye tracing agent can fully infiltrate into the deep soil layers and preclude any potential interference of the frames with the dyeing test. Each dye tracing profile was primarily based on double-ring infiltration, which consisted of two rectangular steel frames, an inner one measuring 120 cm (length) × 60 cm (width) × 50 cm (height) and an outer one measuring 140 cm × 80 cm × 50 cm, concentrically buried in the soil at a depth of 30 cm (Fig 1). After burial, the soil within 5 cm around the frames was compacted to prevent the dye tracing agent from infiltrating along the frames. A sprayer (GP1000, Baoding, China) was fixed above the centre of the frame, controlling the dye release speed with a constant flow pump (BT100-02, Baoding, China). According to local rainfall data, a bright blue dye tracer FCF (4 g L^−1^), equivalent to 60 mm of rainfall, was slowly sprayed onto the soil surface to detect preferential flow paths. During the spraying experiment, water of equal depth was sprayed on the soil between the two frames to prevent lateral infiltration of the bright blue solution.

After 24 h, when the frames were removed, soil disturbance was observed at the edges; hence, only the undisturbed core of the soil was analysed. Two core regions (each measuring 50 cm × 50 cm) were identified. In one core region, 12 horizontal profiles were excavated at a vertical depth of 5 cm, while in the other core region, 5 vertical profiles were excavated at a horizontal width of 10 cm. The maximum excavation depth was 60 cm for both regions. A digital camera (500D, Canon, Japan) was used to vertically capture each profile. Adjacent to each plot, the litter layer was removed, and soil samples of approximately 500 g were collected at 10 cm intervals along the soil depth, in triplicate, to measure parameters such as moisture content, bulk density, total porosity, soil mechanical composition and organic matter content.

### Image analysis

The processing of the stained images was based on computer morphological image processing techniques (Fig 1). Firstly, the stained image dimensions were cropped to 50 cm × 50 cm, equivalent to 500 × 500 pixels, using ARCMAP 10.2 software (Environmental Research Systems Institute, Redlands, CA, USA). Secondly, Adobe Photoshop CS3 (Adobe Systems, San Jose, CA, USA) was used to adjust brightness, contrast, hue, saturation and perform colour replacement to replace the stained area with black and the unstained area with white. Thirdly, Image-Pro Plus 6.0 software was used for noise reduction and image analysis, converting the image to a binary matrix, where the unstained area was represented as white with a value of 255, and the stained area was represented as black with a value of 0. Fourthly, the binary matrix output was analysed for stained feature parameters using Excel and JMP, including preferential flow dye coverage (DC), depth of matrix flow (UF), preferential flow fraction (PF), length index (Li) and maximum infiltration depth (MD). Specific parameter calculation information referenced from the study by Kan et al. [26].

The extraction of characteristic parameters of preferential flow paths utilised the morphological analysis processing-watershed algorithm in Image-Pro Plus 6.0 software. The watershed function was set to divide connected stained areas into independent and closed patches to obtain spatial location coordinates, quantity and area information of the patch centroids. The area of the patches was determined by the number of pixels they occupied, approximating the area as a circle and calculating the impacting radius of the preferential flow paths based on the circular area formula. In this study, the pore size ranges of preferential paths were divided into four categories: 1.0–2.5, 2.5–5.0, 5.0–10.0 and >10.0 mm.

The analysis of spatial patterns was carried out through the application of the O-ring statistics method. This method, which is predicated upon Ripley’s K-function and the mark correlation function, substitutes rings for circles to function as a statistical tool. Thereby, it is capable of eliminating the bias arising from accumulative measures by means of the probability density function. Furthermore, the O-ring statistics method can be implemented using the Programita2014 software, with the spatial location coordinate information of the preferential flow path serving as the initial input data. Univariate point pattern analysis was conducted with 1/5 (100 mm) of the side length of the stained sample plot as the research scale and a step size of 1 mm. For univariate statistics, if the sample points were uniformly distributed, then the complete spatial randomness (CSR) null model was used; if the distribution of sample points showed significant spatial heterogeneity, then the heterogeneous Poisson process (HPP) null model was used. Through 19 Monte Carlo simulations to obtain a 95% confidence interval (i.e. upper and lower envelope traces), if O11(r) value exceeded the upper envelope trace, it indicated an aggregated distribution denoted by a plus sign (+); if the O11(r) value was between the upper and lower envelope traces, it indicated a random distribution denoted by ’r’; and if the O11(r) value was less than the lower envelope trace, it indicated a uniform distribution denoted by a minus sign (-). The formula for univariate statistics was as follows:

$$O_{11}\left(r\right)=\frac{\sum_{i=1}^{n}Points[R_{i}^{w}\left(r\right)]}{\sum_{i=1}^{n}Area[R_{i}^{w}(r)]}$$

In the equation, *O*_11_(*r*) is a univariate O-ring statistic; *n* is the number of preferential flow paths of a certain influence radius within the observed area; Rwi(r) denotes the *i*th point from the centre of the preferential flow path for that influence radius, with *r* as the radius, mm; *w* is the ring width, mm; Points [Rwi(r)] indicates the number of preferential flow paths within the ring for that influence radius; and Area [Rwi(r)] stands for the area of the ring, mm^2^.

In the present study, the JMP13.0 software was employed to perform statistical analyses and graphical representations for the characteristic parameters of soil preferential flow as well as those of the preferential flow path across different slope positions. Moreover, the significance of differences in soil physical properties among distinct slope positions and soil layers was examined through one-way analysis of variance.

## Results

### Soil properties

**Fig 2:**
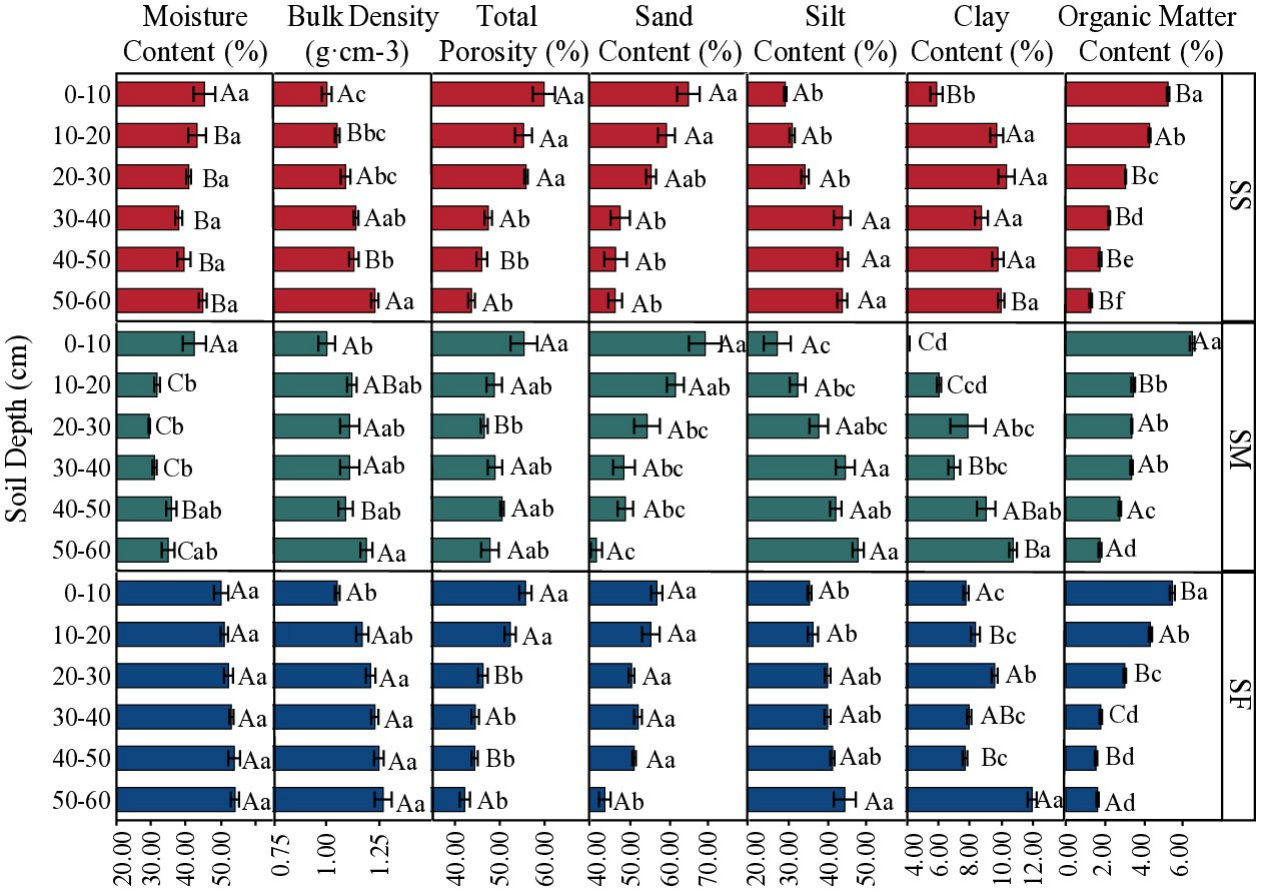
Physical properties of soil. Different capital letters indicate significant differences among different slope positions (p < 0.05); small letters indicate significant differences among different soil layers at the same slope position (p < 0.05).

Different slope positions have significant differences in soil structure characteristics. As shown in Fig 2, different slope positions had a significant impact on soil moisture content, with the trend of SF > SS > SM. The bulk density of SF soil was significantly greater than that of SM and SS (*p* < 0.05), and the organic matter content and soil macroporosity of SM soil were significantly greater than those of SS and SF (*p* < 0.05). Moreover, the sand content of soil showed a trend of SM > SS > SF, while the silt and clay content of soil exhibited a trend of SF > SS > SM. The variations in soil structure characteristics at different slope positions were consistent with the depth of the soil layer, showing that as the soil layer depth increased, the bulk density, silt content and clay content of the soil likewise increased, while the total porosity, macroporosity, organic matter and sand content of the soil decreased.

### Characteristic of preferential flow morphology in the vertical profiles of soil

**Fig 3:**
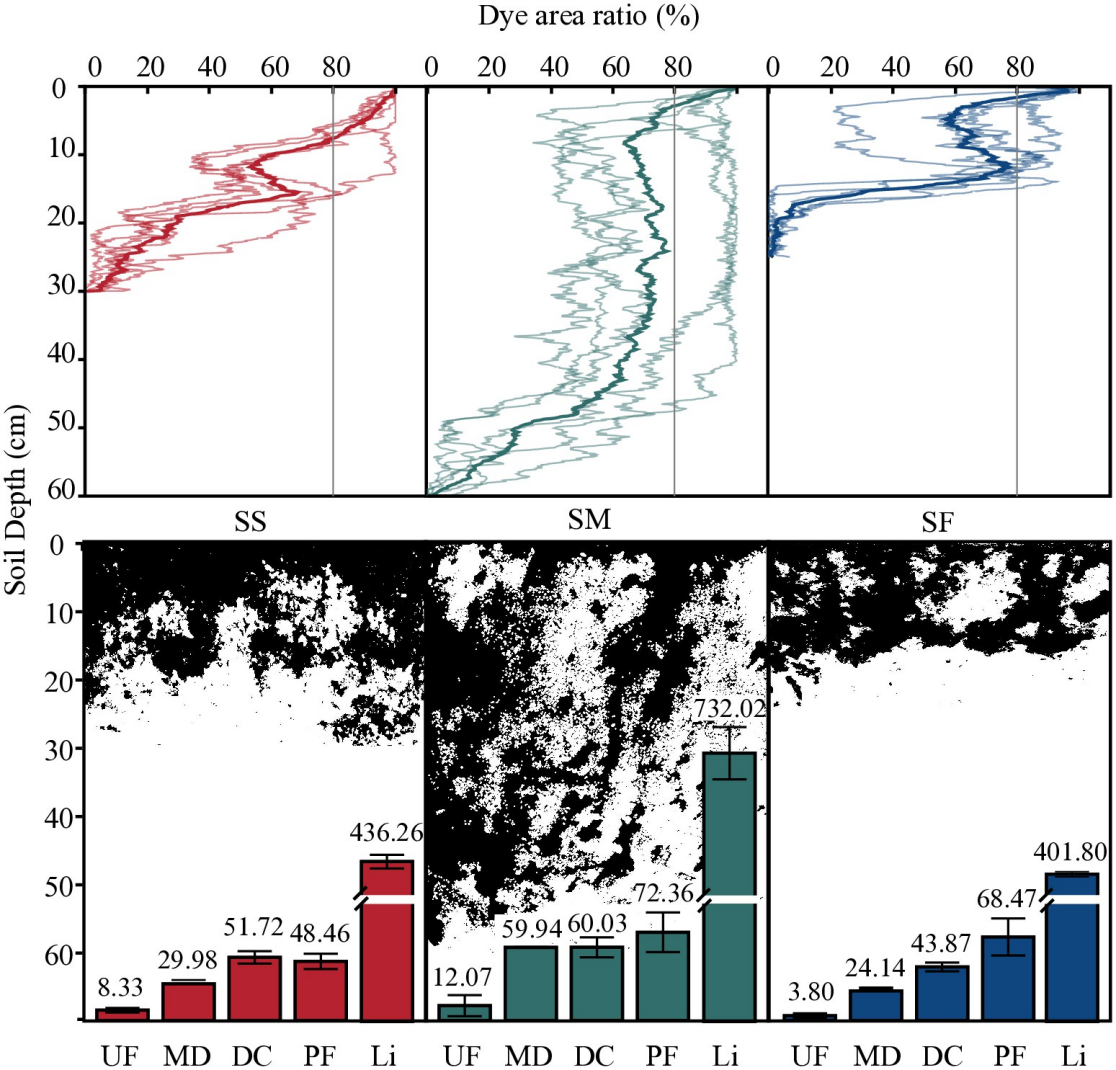
Images of soil dyed vertical profiles and dyed characteristic parameters. UF depth of matrix flow (cm); MD maximum infiltration depth (cm); DC average dye coverage (%); PF preferential flow fraction (%); Li length index (%).

The stained images of soil vertical profiles of the plots at different slope positions all showed varying degrees of staining morphology differentiation, that is, an obvious preferential flow phenomenon was presented (Fig 3). The SS and SF plots exhibited lateral shifts in preferential flow staining morphology, while the SM plot mainly showed vertical development. An analysis of the vertical section staining morphology parameters of different sloping positions shown in Fig 3 reveals that the dye coverage of the vertical section stains of the soil in different slope positions decreased with increasing soil depth. The SS and SF plots showed a similar ‘S’-shaped unimodal trend, with peaks occurring at soil depths of 15.5 and 11.85 cm, respectively, while the SM plot exhibited a ‘J’-shaped trend. The maximum infiltration depths were 59.94 cm in SM, 29.98 cm in SS and 24.14 cm in SF, with significant differences between different sloping positions at the 0.05 level. The average dye coverage, soil matrix flow depth and preferential flow ratio all showed decreasing trends of SM > SS > SF, with no significant differences between different positions (*p* < 0.05). The preferential flow length index of different slope positions showed significant differences (*p* < 0.05), with values of 436.26% in SS, 732.02% in SM and 401.8% in SF.

### Quantities and locations of preferential flow paths

**Fig 4:**
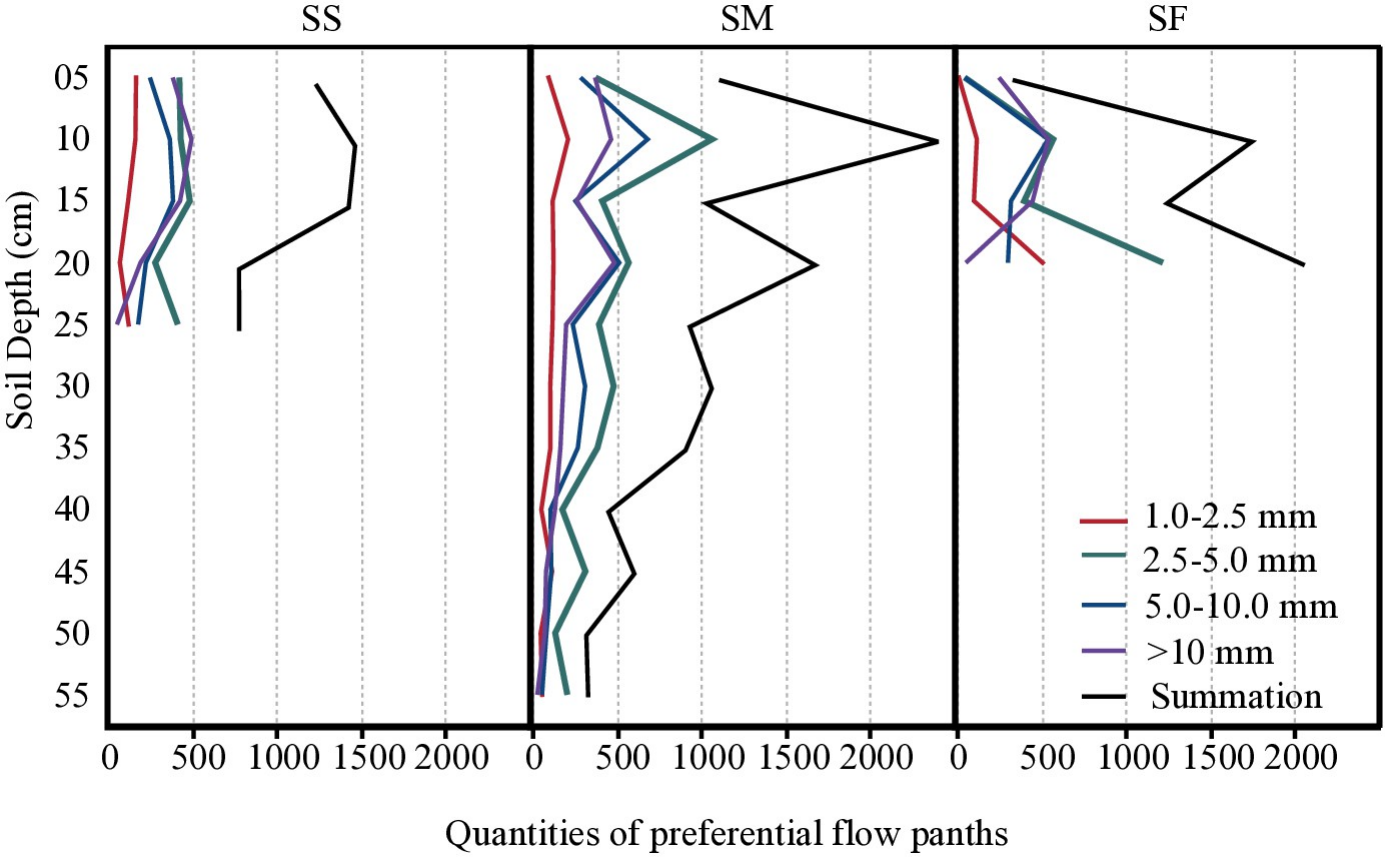
Number of preferential flow paths in different soil layers.

The preferential paths of the horizontal staining profiles in different slope position plots were divided into four pore size ranges according to their influence radii: 1.0–2.5, 2.5–5.0, 5.0–10.0 and >10.0 mm. The quantities and locations of these paths were statistically analysed (Fig 4). However, due to the more than 80% soil dye coverage of the 0–10 cm soil layer, although the matrix flow and preferential flow coexist, their staining morphologies were not distinct, which made it difficult to accurately extract relevant information about the preferential pathways in this soil layer. Therefore, this study only conducted quantitative analysis on the preferential flow paths below 10 cm soil depth. Across the various plots positioned on different slopes, the total number of preferential flow paths followed this order: 2.5–5.0 > 5.0–10.0 > >10.0 > 1.0–2.5 mm. Notably, the highest number of preferential flow paths within each soil layer fell within the pore size range of 2.5–5.0 mm. In the SS, SM and SF plots, the total number of soil preferential flow paths were 4365, 9754 and 5057, respectively. In the SS plot, both the total number of preferential flow paths and the numbers of preferential flow paths in the three pore size grades of 1.0–2.5, 5.0–10.0 and >10.0 mm decreased with the increase of soil depth, and the number of preferential flow paths of 2.5–5.0 mm significantly decreased in the 20 cm soil layer. In the SM plot, both the total number of preferential flow paths and the numbers of preferential flow paths with the same pore size showed a W-shaped small serrated decrease distribution with the increase of soil depth, and the peaks appeared in the 20 30 and 45 cm soil layers. These peaks were mainly affected by the preferential flow paths with the pore size of 2.5–5.0 and 5.0–10.0 mm. In the SF plot, both the total number of preferential flow paths and the numbers of preferential flow paths of 1.0–2.5 and 2.5–5.0 mm showed a V-shaped distribution with the increase of soil depth, presenting as 20 cm > 10 cm > 15 cm. In addition, the numbers of preferential flow paths of 5.0–10.0 and >10.0 mm all showed a decreasing trend with the increase of soil depth.

### Spatial association of preferential flow paths

**Fig 5:**
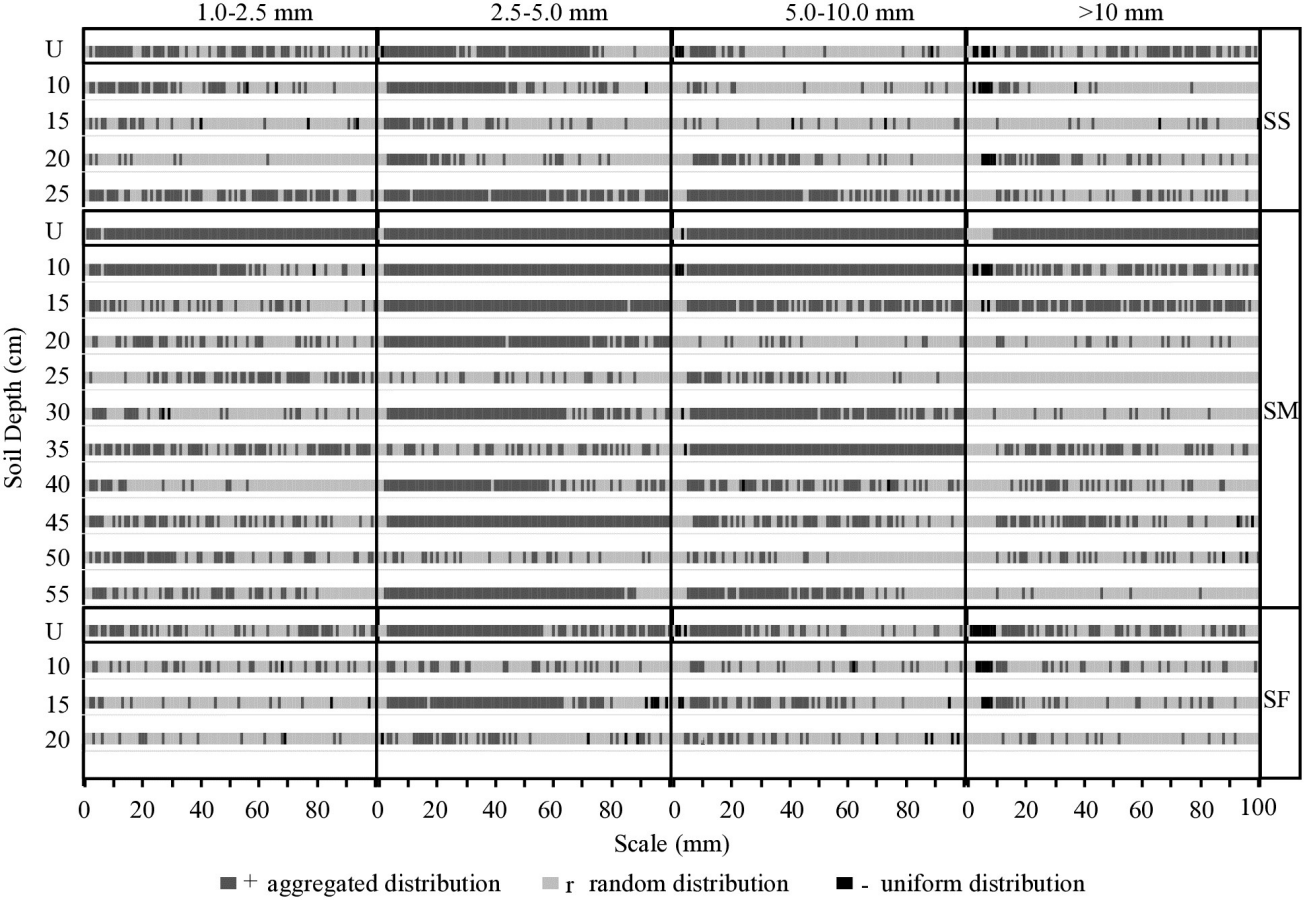
Spatial associations of soil preferential flow paths with different pore size range.

Further analysis was conducted on the spatial distribution of preferential flow paths of different pore sizes in plots at different slope positions (Fig 5). Without considering the variation in soil depth, preferential flow paths of 1.0–2.5, 2.5–5.0 and >10.0 mm in the SS plot were found to be aggregated, while the 5.0–10.0 mm preferential flow path was randomly distributed. In the SM plot, preferential flow paths of all pore sizes were aggregated, whereas in the SF plot, only the 2.5–5.0 mm preferential flow path was aggregated. The spatial distribution of soil preferential flow paths in different slope positions was analysed with the change of soil depth. In the SS plot, the aggregated distribution of the preferential flow path of 2.5–5.0 mm was found to be predominant, and with the increase of the distance scale, the distribution of the preferential paths of each pore size range gradually shifted from aggregated distribution to random distribution. In the SM plot, the 2.5–5.0 and 5.0–10.0 mm preferential flow paths were primarily aggregated, with randomness prevailing only in the 25 and 50 cm soil layers. Additionally, the 1.0–2.5 and >10.0 mm preferential flow paths were mainly aggregated in the 10–15 cm soil layer, while in the SF plot, only the 2.5–5.0 mm preferential flow path was aggregated in the 15 cm soil layer, with the rest being randomly distributed.

## Discussion

### Preferential flow characteristics under different slope positions

The staining characteristic parameters of preferential flow in soil at different slope positions were investigated in this section. Under the same external water supply condition, preferential flows in different slope position plots were found to occur in the topsoil layer at 0–10 cm, and their occurrence times had similarities. However, the degree of differentiation of preferential flow varied with the increase of soil depth. The plot at the middle slope was more likely to generate preferential flow than those at the slope shoulder and the slope foot. Furthermore, the degree of development of preferential flow in the vertical direction in the plot at the middle slope was the highest, and the average maximum infiltration depth at the middle slope was 2 times that at the slope shoulder and 2.5 times that at the slope foot. However, previous studies have found that the degree of development of preferential flow and the spatial non-uniformity of infiltration distribution decreased with the decrease of slope position height [17]. In the present study, the stronger vertical water transport capacity of the plot at the middle slope compared to that at the slope shoulder and the slope foot. This finding not only reveals the intricacy and variability inherent in the impact of slope position on the preferential flow within the soil but also emphasizes the necessity of incorporating the consideration of slope position within a comprehensive framework during the actual processes of soil hydrological research and management.

This difference can be mainly attributed to the following aspects. Firstly, affected by the initial soil water content [27, 28], the plot at the slope foot, which collects runoff, has a relatively high initial soil water content. As a result, the soil water occupies more preferential flow paths, leading to the decrease of the vertical transport capacity of the preferential flow paths. Secondly, soil texture and porosity can reflect that the soil has good structure, ventilation and water permeability, all of which are important factors affecting the vertical transport of preferential flow [29, 30]. In this study, the plot at the middle slope had a smaller soil bulk density and clay content, higher soil porosity and sand content and larger water transport space in the soil layer. The upper incoming water was mainly used to meet the demand of the preferential path to transport water downward, resulting in the enhancement of the vertical transport capacity of the preferential path. Finally, studies have shown that the channels formed by living roots and decayed roots are the main preferential channels that provide a continuous path for macropore flow [31]. The relatively dense shallow root system of grassland also promotes the formation of preferential flow paths in shallow soil and effectively conducts soil water [32]. In the plot at the middle slope in this study, the tree cover is small, the cover of herbs and shrubs is large, and the soil root coverage is sufficiently large to form a relatively dense preferential flow path network, promoting the vertical transport ability and infiltration variability of soil preferential flow [33, 34].

In addition, this study found that preferential flow in the plots at the slope shoulder and the slope foot shows different degrees of lateral transport forms. The peak value and peak range of the lateral transport of preferential flow in the plot at the slope foot are both smaller than those in the plot at the slope shoulder, that is, the occurrence range and development degree of preferential flow in the plot at the slope foot are smaller than those in the plot at the slope shoulder. This result may also be caused by the distribution of the soil root network [35]. The cover of herbs and shrubs in the plots at the slope shoulder and the slope foot is small, and an overall preferential path network has not been formed. Additionally, only a preferential flow path network is formed in a small range where the roots are aggregated, resulting in the lateral transport of preferential flow. The cover of herbs in the plot at the slope shoulder is also larger than that in the plot at the slope foot, and the cover of shrubs is smaller, so the lateral transport is formed in a deeper soil layer. The above results show that the slope position is an important factor affecting the occurrence and development of soil preferential flow. The variability of factors such as soil properties, water content, and vegetation types in different slope positions makes the degree of development and spatial distribution of soil preferential flow differentiated, thereby promoting the flow and redistribution of soil water in the vertical and horizontal directions and realising the diversification of water storage and utilisation.

### Spatial distribution of preferential flow paths under different slope positions

An in-depth analysis of the quantity and spatial structure status of the preferential flow paths in the soil reveals significant differences in the quantity of preferential paths with various pore sizes among the sample plots at different slope positions. Nonetheless, the total distribution of preferential paths with each pore size is the same. The number of preferential flow paths with 2.5–5.0 mm in each soil layer was the largest and exhibited an aggregated distribution. This result indicates that although the soil environments at different slope positions are different, the magnitude relationship of the quantity of preferential flow paths corresponding to each pore size is consistent. The preferential flow transport in the soil is mainly dominated by the preferential paths with a large number of small pore sizes. The preferential flow paths of these small pore sizes exhibited aggregated distribution and strong vertical connectivity. This result is related to the quantity of plant roots of different diameter classes in the soil. In the stratified investigation of plant roots in the sample plots, most of the plant roots in the sample plots were within the 0–3 mm range. Cai et al. [36] discovered a significant promotion effect of plant roots ranging from 2.0 mm to 5.0 mm on preferential flow in soil, while plant roots <2 mm had no impact on it. Additionally, with increasing distance scales on different slopes, the 2.5–5.0 mm preferential flow paths shift from aggregated to random distribution patterns, which also proves that the 2.5–5.0 mm preferential flow paths are primarily formed by root network systems. Secondly, the quantity of the preferential paths with 1.0–2.5 mm and 2.5–5.0 mm has the same changing trend, indicating that a larger infiltration head may cause the 1.0–2.5 mm path to transform into a 2.5–5.0 mm path [37].

In the vertical direction, as the soil depth increases, the total quantity of preferential flow paths in the soil of sample plots at different slope positions and the quantity of preferential flow paths with the same pore size generally show a decreasing trend with the increase of soil depth [38]. Affected by human disturbance and the composition of soil particles, the surface soil has a smaller bulk density, loose soil texture, more macropores, and a larger quantity of plant roots and degree of biological activity; thus, the quantity of preferential paths in the surface soil is relatively large [39]. Remarkably, on slope shoulder plots, the preferential flow paths of 1.0–2.5, 2.5–5.0 and >5.0–10.0 mm showed an aggregated distribution pattern at the 25 cm soil layer. However, in other plots, the spatial correlation of preferential flow paths with increasing soil layer depth showed no clear pattern. This finding indicates that the spatial correlation of preferential flow paths is unrelated to changes in soil layer depth but rather to the distribution of plant roots. The quantity of preferential flow paths for each pore size in the middle-slope sample plots decreases in a W-shaped small serrated distribution with the increase of soil depth and exhibited aggregated distribution pattern. This result indicates that compared to other sample plots, the spatial distribution variability of preferential paths in the middle-slope sample plots is stronger and the preferential flow differentiation is more intense. The soil layer where the peak of the quantity of preferential paths appears is consistent with the peak of the staining area ratio, indicating that the preferential paths are an important way for soil water transport.

The above results indicate that the formation and development of preferential flow are directly influenced by the number, composition and distribution of preferential flow paths. Different slope positions have soil preferential path networks that are affected by root distribution and soil texture, leading to significant variations in their aggregation and connectivity. This results in differing contributions of preferential flow to the soil hydrological process. Therefore, understanding the spatial distribution patterns of preferential flow path networks in different slope positions is crucial for accurately simulating and predicting soil hydrological processes. However, in the present study, the intricate interplay between the slope position and other factors exerting an impact on soil preferential flow remained inadequately probed. Future endeavors could involve the establishment of more sophisticated models or the conduct of long-term monitoring experiments, thereby enabling an accurate apprehension of the influence of such interactions on the formation mechanism of soil preferential flow.

## Conclusion

This study explored the impact of slope position on the characteristics of soil preferential flow and the spatial distribution features of soil preferential flow paths within the forested area of Simian Mountain. It was observed that a highly differentiated preferential flow phenomenon prevailed in the middle of the slope. Notably, factors such as soil properties, soil moisture content, and soil vegetation types across different slopes emerged as crucial determinants influencing the occurrence and development of soil preferential flow. The migration of preferential flow at various slope positions predominantly took place through the preferential flow path with a pore size ranging from 2.5 to 5.0 mm. Moreover, the aggregated distribution of these preferential paths constituted a necessary prerequisite for the initiation and development of preferential flow. The aforementioned results indicate that the spatial heterogeneity of the preferential flow path is subject to the influence of slope position, and consequently, when investigating soil hydrological processes involving preferential flow, the impact of slope position must be duly considered. Secondly, the spatial distribution network of the preferential flow path serves as the principal conduit for the occurrence and development of preferential flow in soil. The spatial distribution characteristics of priority flow paths in soil at different slopes can function as an important index for quantifying and predicting the formation mechanism of priority flow in soil. This, in turn, can offer a novel approach for probing the contribution of preferential flow to soil hydrological processes and furnish a scientific foundation for the management of regional water resources and the restoration of vegetation cover.
